# The Effect of Recovery Duration on Technical Proficiency during Small Sided Games of Football

**DOI:** 10.3390/sports4030039

**Published:** 2016-07-08

**Authors:** Scott McLean, Hugo Kerhervé, Mitchell Naughton, Geoff P. Lovell, Adam D. Gorman, Colin Solomon

**Affiliations:** 1 School of Health and Sport Sciences, University of the Sunshine Coast, Sippy Downs, QLD 4556, Australia; hugokerherve@gmail.com (H.K.); mrn004@student.usc.edu.au (M.N.); agorman@usc.edu.au (A.D.G.); csolomon@usc.edu.au (C.S.); 2 School of Social Sciences, University of the Sunshine Coast, Sippy Downs, QLD 4556, Australia; glovell@usc.edu.au

**Keywords:** small sided games, football, recovery, technical skill, pacing

## Abstract

The aim of this study was to determine the effect of increasing the duration of the recovery periods separating serial bouts of small sided games (SSG) of football on technical skills (TS). Twelve semi-professional footballers (mean ± SD; age 21 ± 3 years; VO_2peak_ 64 ± 7 mL∙min∙kg^−1^; playing experience 15 ± 3 years) completed two SSG sessions, consisting of 3 vs. 3 players and 6 bouts of 2 min, separated by either 30 s recovery (REC-30) or 120 s recovery (REC-120). Sixteen TS, including passing, possession, and defensive related variables, and exercise intensity (heart rate, rating of perceived exertion, time motion descriptors) during the bouts were measured. Repeated measures ANOVA were used to determine differences between-conditions, for TS. The number of successful tackles was significantly higher, and the average time each team maintained possession was significantly lower in REC-120 compared to REC-30. There were no significant differences for all other TS variables, or exercise intensity measures between REC-30 and REC-120. Overall, a four-fold increase in the duration of recovery separating SSG bouts did not alter the technical skill execution of players. The experience and skill level of the players, combined with an apparent regulation of effort through pacing, may have assisted in the maintenance of technical skill execution.

## 1. Introduction

The execution of technical skills (TS) is the fundamental component of football [1,2]. Players must apply cognitive, perceptual, and motor skills in a process where decisions are made and executed in a rapidly changing environment [3,4,5]. Ultimately, the successful execution of TS, including passing, tackling, dribbling, and shooting, are likely to be primary determinants in the outcome of a match [2,3,6].

Successful execution of TS by football players could be decreased by the fatigue that occurs during a match [2,7]. Players experience temporary fatigue following phases of high intensity exercise during a match, and progressive fatigue across the duration of a match [8]. The mechanisms responsible for the accumulation of fatigue in players during match-play are varied, complex and not completely understood [9,10]. Depletion of energetic substrates (e.g., muscle glycogen, muscle creatine phosphate), increased metabolic by-products (e.g., lactate, potassium), increased pH, and dehydration, have all been proposed to contribute to the accumulation of fatigue during a football match [10]. Despite the lack of a definitive conclusion regarding the mechanisms of fatigue during a match, it is implied that a decline in TS in the later stages of a match is attributable to fatigue [9,11].

Investigations into the relationship between fatigue, TS, and decision making are often conducted using non-football match specific or laboratory-based football simulations [4,9,12,13]. Additionally, non-match specific simulations do not account for factors such as player interactions, field positioning, and match related stressors both physiological and psychological. Furthermore, the participants do not experience the same sensory state as in an actual match, therefore reducing arousal levels, which can influence playing performance [14]. Small sided games (SSG), although different to actual match-play, combine the physiological, technical, tactical, and decision making components of football [15,16,17], thereby providing a more representative test environment compared to non-match play and laboratory based studies. The SSG format is also an effective training method, using conditions that are representative of match demands [18]. Typically, SSG are used in an interval format, consisting of a series of bouts and recovery periods [19,20,21,22].

Progressive increases in heart rate (HR), rating of perceived exertion (RPE), blood lactate [La], and decreased high intensity running during SSG bouts have been associated with decreases in TS [19,20,23,24]. However, increasing the duration of recovery during repeated bouts of high intensity exercise increases physiological recovery and may allow physical performance to be maintained [25]. Mclean et al. (2016) have shown that increasing the duration of the recovery period from 30 s to 120 s separating serial SSG bouts, significantly increased physiological recovery, both systemically (HR), and locally (vastus lateralis muscle oxygenation, using near infrared spectroscopy) in experienced semi-professional players, [26].

Only one study has investigated the effect of the duration of the recovery period during SSG on TS, using recovery durations of 1, 2, 3, and 4 min separating the bouts [24]. The increased recovery duration produced an increased number of total and successful passes, tackles, and passes received, suggesting that the longer recovery duration may have decreased progressive fatigue and allowed the TS to be maintained during the SSG [24]. Increasing the duration of recovery periods separating serial bouts may reduce physiological and psychological stress in subsequent bouts, allowing players to maintain optimal arousal levels and the subsequent attentional levels required for successful TS execution [4,14]. Exercising at very high levels of intensity elevates arousal levels, and has a negative impact on attentional processes, resulting in players missing the important cues that are necessary for successful skill execution [4].

Despite only measuring five technical variables, Koklu et al. (2015) found that a shorter recovery duration decreased TS in youth participants [24]. To obtain a more comprehensive understanding of the effect of recovery duration separating SSG bouts on TS, it is necessary to assess additional TS variables, and use experienced players. Analyzing additional (to previously measured) technical variables, including first touch passing, the number of individual touches occurring during each possession, and time in possession for individuals and teams, will provide necessary information to establish whether TS execution is affected when the recovery periods separating SSG bouts are manipulated. First touch passing involves high technical difficulty and information processing to recognize the positioning of team members and defenders [20]. Determining the number of touches a player selects per possession, and the duration of time that each individual possesses the ball during SSG, will provide additional information on whether the technical components of football are influenced by fatigue.

Therefore, the aim of this study was to determine whether changing the recovery duration during the same 3 vs. 3 SSG format would have an effect on multiple, specific individual, and team TS variables. It was hypothesized that during serial bouts of SSG, a 120 s recovery period compared to a 30 s recovery period, would allow players to maintain their TS execution.

## 2. Materials and Methods

### 2.1. Participants 

Twelve experienced male semi-professional football players (mean ± SD; age 21 ± 3 years; VO_2peak_ 64 ± 7 mL∙min∙kg^−1^; playing experience 15 ± 3 years) [26], playing in the same team and competing in the second tier of football in Australia, participated in the study. Participants trained three times per week for an approximate weekly total of 240 min and competed in one match per week of 90 min duration. The study received institutional ethical approval and participants were informed of the study requirements and provided written informed consent.

### 2.2. Study Design

This study was conducted using a one-group, repeated measures design. The independent variables were the two different recovery durations, 30-s recovery (REC-30), and 120-s recovery (REC-120), and the number of bouts (1–6). The dependent variables were the individual and team technical skill variables. Participants completed the SSG sessions, and a peak aerobic capacity test (laboratory conditions). All testing and data collection was completed in an 11-week period during the participants’ competitive season. The exercise intensity during the bouts of the SSG was monitored using heart rate (HR), rating of perceived exertion (RPE; Borg CR-10 scale), time motion descriptors (TMD) (speed and distance), as previously reported (see McLean et al., 2016, for the methods used for these measurements) [26]. In the current original sub-set of exercise intensity data there was a significant (*p* < 0.05) decrease for HR in REC-120 compared to REC-30 during the recovery periods. There were no significant (*p* > 0.05) differences for HR, RPE, or TMD between REC-30 and REC-120 during the bouts.

### 2.3. Small Sided Games

Each participant completed REC-30 and REC-120 under the same SSG format consisting of 3 vs. 3 players with 6 × 2 min bouts played on a 15 m × 20 m natural grass pitch [26]. Participants were tested in two separate sessions, separated by a minimum of 2 days and maximum of 5 days, and the order of conditions was counterbalanced. The SSG testing was completed in 8 sessions during a 5-week period. The SSG testing sessions were completed at the beginning of the participants’ normal football training session and at the same time of day to avoid variations to circadian rhythm. The SSG teams were selected by the two experienced team coaches to ensure that similar levels of technical ability and physical capacity of players were evenly distributed across the two teams. The same team membership was used for all testing sessions. The objective of the SSG was to maintain possession as a team, with unlimited ball contacts per possession, with no goals or goalkeepers, and no coach encouragement. Additional balls were placed around the pitch to minimize time lost for balls out of play. Prior to testing, participants performed a standardized warm up. For the recovery periods, participants were instructed that they could walk within the playing area.

### 2.4. Technical Skills Analysis

The SSG were recorded using a high definition video recorder (Sony HDR-CX130) positioned on a tripod at approximately 3 m above the playing surface, at the center of the 20 m length of the playing area and approximately 10 m from the boundary of the playing area. Post hoc analysis of the video recordings was used to analyze the TS of participants. Technical actions were divided into 16 different categories (refer to Table 1 for definitions). Intra and inter-observer (2 observers) reliability were assessed by analysis of three randomly selected bouts. An observation by observation breakdown of the coding results was used for statistical analysis [19]. Intra- class correlations showed a high degree of intra-observer reliability (ICC > 0.801), as well as high inter-observer reliability using Cohen’s Kappa (*k* > 0.814). The reliability results therefore indicated a very high level of agreement [19].

### 2.5. Statistical Analysis

Statistical power calculations (using G * Power) using the mean difference and standard deviation in the variable with the largest difference in mean values between conditions, average team possessions, was low (0.26). However, despite the low power we were able to detect a significant difference in two of the TS dependent variables, which indicates that the design and number of participants was satisfactory. Statistical analyses was performed using SPSS (version 22, IBM Corporation, Armonk, NY, USA). A two-way repeated-measures ANOVA was used for each dependant variable to assess the effect of condition (REC-120 and REC-30), bout (1-6), and condition × bout interactions for all dependent variables. Assumptions of sphericity using Mauchly’s W test were used, with adjustments for significant results implemented using the Greenhouse-Geisser correction of degrees of freedom. Post hoc pairwise comparisons were used to investigate significant interactions, and were conducted using the least significant difference (LSD) method with no adjustments for multiple comparisons. A paired *t*-test was used to compare the total distances covered between REC-30 and REC-120, in the SSG sessions. Partial eta-squared (ɳ_Ƥ_^2^), was used as an indicator of effect size, (range was small 0.01, medium 0.06, and large 0.14) [27], and statistical power were reported. Data are reported using mean ± SD, and the level of significance was set at *p* < 0.05.

## 3. Results

### Technical Skill

For TS there was a significant main effect of condition with a large effect size for the number of successful tackles (*p* = 0.023; ɳ_Ƥ_^2^= 0.071; β = 0.313), with an increased number of tackles performed in REC-120. There was also a significant main effect and large effect size for the average time of team possessions (*p* = 0.038; ɳ_Ƥ_^2^ = 0.807; β = 0.660), with possessions being lower in REC-120. There were no significant (all *p* values > 0.05) main effects for condition, or bout number, and no condition × bout interaction, for all other technical variables (Table 2 and Table 3).

## 4. Discussion

This study was designed to determine the effect of increasing the recovery duration between serial bouts of SSG from 30 s to 120 s on a set of TS in experienced participants. Contrary to the hypothesis, the main result was that overall TS execution was not influenced by the increased recovery duration. From the TS that were analyzed in the current study, only two out of the sixteen variables were significantly different (and with large effect sizes): duration of team possessions (shorter), and the number of successful tackles (higher) in REC-120 compared to REC-30. The shorter duration of possessions measured in REC-120 may be due to the increased number of tackles performed in that condition. It is possible that the increased physiological recovery (HR) during REC-120 allowed the players to perform more tackles, subsequently reducing time in possession. The absence of any significant differences, between the conditions or across the bouts, for all other TS variables needs to be considered in the context of the low statistical power in the study design Nevertheless, the consistency of the means between the conditions and across the bouts indicates that practical differences were unlikely. The absence of an overall difference between conditions in TS occurred despite a significantly decreased HR, during the recovery periods in REC-120 compared to REC-30.

The current result is in contrast with those of the only other study that has investigated TS execution when using different recovery durations separating SSG bouts, in which there was an increase in total passes, successful passes, passes received, and tackles during bouts interspersed with recovery durations of 3 and 4 min, compared to the 1 min [24]. In the study of Koklu et al. [24], the HR in the 3 and 4 min recovery conditions were significantly lower than in 1 min recovery condition, indicating an increased physiological recovery, which may have affected performance of TS [24]. In the current study, TS was not affected with an increase in the duration of the recovery period. A possible explanation is that highly trained and skilled athletes are able to adapt to the demands to which they are exposed, including the maintenance of skill execution while experiencing fatigue [14]. In support of the current results, no declines were observed in the TS execution of professional midfield players during full-scale matches [11]. This was despite players experiencing end-game fatigue, as indicated by significant decreases in high speed running in the final 30 min of the game [11].

One of the critical attributes of skilled performers is their capability to continually adapt to the demands of the performance context [28,29,30]. Skilled athletes can compensate for changes in the performance environment to maintain consistent performance outcomes [14]. This is a potential explanation for the maintenance of TS between REC-30 and REC-120 in the current study. A further facilitator for the maintenance of TS, could be explained by the players regulating effort [26]. The exercise intensity measures in the current study were not different between the SSG conditions (REC-30, REC-120), indicating that the exercise intensity level and work performed during the SSG were similar for both of these conditions [26]. The ability to regulate effort to the appropriate level is increased when individuals are highly trained, have task-specific experience, and possess knowledge of the exercise endpoint [31,32,33,34,35]. The experience level (14.6 years), aerobic capacity (VO_2peak_ of 64 mL∙min∙kg^−1^), experience in SSG (a regular component of training) of the participants, and known exercise endpoint, suggests that the current participants were well equipped to be able to regulate their effort to maintain their TS.

In addition to investigating individual TS variables, we measured the TS performed per minute, which allowed comparisons to the frequency of TS performed during matches. One study has reported that elite players were involved with the ball on approximately 45 occasions during a 90 min match, which equates to two ball related actions per minute [36]. In the current study, the TS performed per minute of the SSG was not significantly different between conditions, despite being almost double (REC-30 = 3.6, and REC-120 = 3.7) the TS performed per minute of a match [36].

Normalizing TS variables as a percentage of totals allows comparisons of specific TS variables across different SSG formats. Successful passing is a commonly used variable that is expressed as a percentage of total passes [20,37]. In the current study, successful passing was 80% (REC-30), and 77% (REC-120), and did not decline across the subsequent bouts. This is contrary to other 3 vs. 3 SSG formats with unlimited ball touches per possession [20], where the percentage of successful passes significantly decreased from bout 1 (76%) to the fourth and final bout (70%), and HR was significantly higher in the fourth bout compared to the first bout [20]. In the current study, the exercise intensity of participants (HR, RPE and TMD) was maintained across the bouts, which may have prevented declines in physical and cognitive performance, allowing TS execution to remain relatively constant across the multiple bouts.

In addition to previously measured TS variables, this study analyzed four TS variables not previously measured in SSG research. The findings of the current study that could assist with the planning of SSG training, are that players utilized three ball touches, with a possession duration of 2.2 s (REC-30), and 2.0 s (REC-120), when instructed to play with unlimited ball touches per individual possession. This is higher than the mean number of touches taken during regular professional matches (Defenders = 1.74, Attacking Midfielders = 2.26) [20], which could be explained by the increased number of passing options available during a normal game. For successful first touch passes there was no significant difference between the two conditions. However, successful first touch passes produced approximately 50% of total successful passes in both conditions. First touch passing tends to increase the pace of the game and requires high levels of information processing, both of which are characteristics of elite level football [20]. The high percentage of successful first touch passing in the current study indicates that SSG played with 3 vs. 3 could provide an appropriate stimulus to develop the information processing required for performance in the normal competitive environment.

Limitations of the current study include that a measure of pacing was not included in the current design, as a measure of pacing could have directly indicated if pacing was consciously used. It is suggested that future research could include objective and subjective measures of pacing to determine whether this strategy is a fundamental factor in maintaining TS while potentially experiencing fatigue. In addition, only one format of SSG was used, and so manipulation of variables such as modifying player numbers, pitch size, number of bouts and recovery periods, or including goals and goalkeepers may have provided further information of the effect of altering recovery duration on different SSG formats.

## 5. Conclusions

It is concluded that increasing the duration of the recovery period separating serial SSG bouts from 30 s to 120 s did not affect the TS execution of experienced and trained football players. It is likely that the trained football players were able to adapt to the increased physical demands of the shorter recovery duration to maintain TS. This is likely due to pacing, whereby players regulate their effort to maintain performance. During a 3 vs. 3 SSG format, experienced and trained football players prefer to take three touches of the ball while in possession, use a high number of first touch passing, and maintain possession for approximately 2 s. Future research in SSG could include the technical actions performed per minute, as in the current study, so that comparisons can be made between studies that utilize different SSG formats. Furthermore, future SSG research could determine the repeat sprint ability of participants as an additional exercise specific indicator of exercise capacity.

## 6. Practical implications

Planning SSG training with 30 s recovery periods separating bouts may provide a more time efficient training session, without compromising the technical skill execution of players. Further, coaches should consider playing experience, and training status of players when designing SSG training sessions, as these will influence the SSG training outcomes. In addition, coaches often impose restrictions on players’ touches in training to encourage playing with fewer touches. In the current SSG format, players chose to take three touches, and to spend approximately 2 s in possession.

## Figures and Tables

**Table 1:** Technical skill definitions.

Technical Action	Definition
Time (s) in possession (individual)	The amount of time (s) an individual player has possession.
Touches in possession (individual)	The number of occasions an individual player has contact with the ball (using the foot, thigh, head, or chest) per possession.
Time (s) in possession (team)	The amount of time (s) a team has possession.
Successful team passes	The number of passes each team completes for each possession.
Successful pass (%)	The percentage of successful passes completed during the bouts (both teams).
Intercept	A player from the non-possession team gains possession by intercepting a pass from the team in possession.
Deflection	A player is struck with the ball, without attempting to play at it resulting in a change/no change of team possession, or the ball goes out of play.
Unsuccessful pass	An incomplete attempted pass between players of same team.
Successful pass	The completion of an attempted pass between players of the same team.
Unsuccessful 1st touch pass	The incompletion of an attempted 1st touch pass between players of same team.
Successful 1st touch pass	The completion of an attempted 1st touch pass between players of the same team.
Successful tackle	A player from the non-possession team involved in a duel gains possession of the ball.
Unsuccessful tackle	A player in possession remains in possession after a duel.
Lost possession (miscontrol)	A player loses ball possession due to miscontrol.
Total possessions per bout	The number of individual possessions, during the bouts (all players).
Technical actions per minute	The sum of successful and unsuccessful passes, successful and unsuccessful first touch passes, successful and unsuccessful tackles, and intercepts performed per minute of the bouts.
Time (s) ball is out of play	Time from when the ball leaves the playing area until a new ball is played back into the playing area.

Time in possession (individual) was recorded when a player first touched the ball until that player’s final touch of the ball (from either a pass, lost possession, or tackle). Time in possession (team) was recorded from the first touch by the player in a passing sequence, to the final touch of the last player in the passing sequence. Deflections are described as an unintentional act, and are therefore not included in the technical actions per minute variable.

**Table 2:** Mean ± SD of technical actions.

Variable	Condition	B1	B2	B3	B4	B5	B6	SSG Session
Time in possession (s)	REC-30 REC-120	2.2 ± 1.0 2.1 ± 1.1	2.1 ± 0.9 1.9 ± 1.3	2.1 ± 1.2 1.8 ± 0.8	1.9 ± 0.8 1.9 ± 0.9	2.2 ± 1.1 2.1 ± 0.9	2.7 ± 3.2 2.3 ± 1.2	2.2 ± 1.3 2.0 ± 1.0
Touches in possession	REC-30 REC-120	3.3 ± 0.9 3.3 ± 1.4	3.4 ± 0.9 3.1 ± 1.3	3.3 ± 1.0 3.0 ± 0.8	2.8 ± 0.5 3.2 ± 0.6	3.4 ± 1.2 3.4 ± 1.1	4.1 ± 2.3 3.6 ± 1.5	3.4 ± 1.3 3.3 ± 1.1
* Average team possession (s)	REC-30 REC-120	7.3 ± 3.4 5.9 ± 2.0	9.3 ± 2.6 7.0 ± 2.7	6.8 ± 1.9 6.5 ± 2.6	7.9 ± 1.8 6.9 ± 4.5	6.7 ± 2.0 6.5 ± 1.6	9.8 ± 6.9 7.0 ± 1.9	7.9 ± 3.4 6.6 ± 2.4
Pass/possession (team)	REC-30 REC-120	1.8 ± 1.1 1.4 ± 0.5	2.8 ± 0.3 1.9 ± 1.1	1.9 ± 0.5 2.4 ± 1.2	3.0 ± 2.0 3.5 ± 3.4	1.6 ± 0.3 1.4 ± 0.3	2.9 ± 2.7 1.7 ± 0.9	3.2 ± 1.4 2.0 ± 1.6
Successful pass (%)	REC-30 REC-120	79 ± 20 71 ± 29	85 ± 12 81 ± 16	84 ± 18 81 ± 18	82 ± 15 77 ± 23	82 ± 25 79 ± 23	80 ± 15 76 ± 17	80 ± 15 77 ± 21
Interceptions	REC-30 REC-120	0.4 ± 0.7 0.8 ± 0.8	0.4 ± 0.7 0.6 ± 0.7	0.3 ± 0.7 0.3 ± 0.5	0.4 ± 0.7 0.3 ± 0.5	0.4 ± 0.5 0.7 ± 0.9	0.2 ± 0.4 0.1 ± 0.3	0.4 ± 0.6 0.5 ± 0.7
Deflections	REC-30 REC-120	0.0 ± 0.0 0.5 ± 1.2	0.2 ± 0.4 0.5 ± 0.5	0.0 ± 0.0 0.4 ± 0.7	0.2 ± 0.4 0.0 ± 0.0	0.3 ± 0.7 0.3 ± 0.5	0.3 ± 0.5 0.3 ± 0.6	0.2 ± 0.4 0.3 ± 0.7
Unsuccessful pass	REC-30 REC-120	0.9 ± 0.7 1.2 ± 1.1	0.4 ± 0.5 0.7 ± 0.7	0.4 ± 0.5 0.8 ± 0.9	0.9 ± 0.8 1.3 ± 1.9	0.6 ± 0.9 0.9 ± 1.0	0.8 ± 1.0 0.8 ± 1.1	0.7 ± 0.8 0.9 ± 1.1
Successful pass	REC-30 REC-120	3.2 ± 2.3 2.4 ± 1.6	4.0 ± 1.7 2.9 ± 1.8	2.8 ± 1.5 3.1 ± 1.4	3.2 ± 1.3 2.6 ± 1.0	2.4 ± 1.6 2.8 ± 1.4	3.1 ± 2.4 3.3 ± 1.5	3.1 ± 1.8 2.8 ± 1.4
Unsuccessful 1st touch pass	REC-30 REC-120	0.1 ± 0.3 0.4 ± 0.5	0.5 ± 0.8 0.3 ± 0.5	0.3 ± 0.5 0.3 ± 0.7	0.3 ± 0.5 0.3 ± 0.7	0.2 ± 0.4 0.3 ± 0.9	0.4 ± 0.9 0.7 ± 0.5	0.3 ± 0.6 0.4 ± 0.6
Successful 1st touch pass	REC-30 REC-120	1.5 ± 1.3 1.5 ± 1.6	1.7 ± 1.3 1.7 ± 1.3	1.1 ± 0.9 1.3 ± 1.0	1.9 ± 1.5 1.2 ± 1.0	1.3 ± 0.8 1.2 ± 0.9	2.0 ± 1.0 1.3 ± 0.8	1.6 ± 1.2 1.4 ± 1.1
* Successful tackle	REC-30 REC-120	0.5 ± 0.7 0.6 ± 0.7	0.3 ± 0.5 1.1 ± 1.1	0.9 ± 0.8 1.3 ± 1.2	0.6 ± 0.7 0.9 ± 0.7	0.8 ± 1.0 0.5 ± 0.7	0.3 ± 0.8 0.7 ± 0.7	0.6 ± 0.8 1.0 ± 0.9
Unsuccessful tackle	REC-30 REC-120	0.8 ± 1.1 0.8 ± 0.8	0.6 ± 0.7 0.9 ± 1.0	0.9 ± 0.9 0.7 ± 1.0	0.8 ± 0.7 0.8 ± 1.0	0.8 ± 1.1 0.3 ± 0.5	0.3 ± 0.5 0.4 ± 0.7	0.7 ± 0.8 0.6 ± 0.8
Technical actions·min^−1^	REC-30 REC-120	3.7 ± 1.4 3.7 ± 1.6	3.9 ± 1.2 4.1 ± 1.4	3.4 ± 1.1 3.9 ± 1.6	4.0 ± 1.3 3.7 ± 1.0	3.3 ± 1.3 3.4 ± 1.1	3.5 ± 1.4 3.6 ± 1.0	3.6 ± 1.3 3.7 ± 1.3
Lost possession (miscontrol)	REC-30 REC-120	0.5 ± 0.7 0.9 ± 12	0.3 ± 0.5 0.5 ± 0.8	0.4 ± 0.7 0.9 ± 1.1	0.3 ± 0.5 0.3 ± 0.5	0.7 ± 0.9 0.4 ± 0.9	0.2 ± 0.4 0.5 ± 0.5	0.4 ± 0.6 1.0 ± 0.9
Total possession per bout	REC-30 REC-120	7.4 ± 3.0 6.9 ± 2.3	7.5 ± 2.5 7.0 ± 2.7	5.4 ± 1.6 6.8 ± 2.6	7.5 ± 2.3 6.3 ± 1.9	5.8 ± 1.9 6.7 ± 1.2	7.1 ± 2.4 7.3 ± 2.5	6.8 ± 2.4 6.8 ± 2.2

* Significant (*p* < 0.05) main effect of condition.

**Table 3:** Probability (*p*), F statistic (*F*), Observed power (β), Partial eta squared (effect size ɳ_Ƥ_^2^) of technical variables for Condition, Bout, and Condition × Bout interaction.

Technical Variable	Condition				Bout				Interaction			
	*p*	*F*	β	ɳ_Ƥ_^2^	*p*	F	β	ɳ_Ƥ_^2^	*p*	*F*	β	ɳ_Ƥ_^2^
Time in possession (s)	0.495	0.497	0.099	0.043	0.368	1.106	0.364	0.091	0.881	0.193	0.080	0.881
Touches in possession	0.672	0.189	0.068	0.017	0.099	1.961	0.617	0.151	0.586	0.617	0.154	0.053
Average team possession (s)	0.038 *	12.51	0.660	0.807	0.424	1.053	0.274	0.260	0.908	0.296	0.102	0.090
Pass/possession (team)	0.557	0.434	0.077	0.126	0.510	0.893	0.235	0.229	0.348	1.218	0.316	0.289
Successful pass (%)	0.305	1.157	0.166	0.095	0.710	0.586	0.199	0.051	0.992	0.099	0.070	0.009
Interceptions	0.223	1.669	0.219	0.132	0.089	2.028	0.634	0.156	0.602	0.732	0.244	0.062
Deflections	0.078	3.786	0.427	0.256	0.698	0.603	0.204	0.052	0.163	1.646	0.530	0.163
Unsuccessful pass	0.226	1.647	0.217	0.217	0.183	1.572	0.509	.125	0.996	0.071	0.064	0.006
Successful pass	0.523	0.435	0.093	0.038	0.330	1.181	0.388	0.097	0.246	1.465	0.327	0.118
Unsuccessful 1st touch pass	0.339	1.000	0.150	0.083	0.652	0.664	0.223	0.057	0.502	0.718	0.190	0.006
Successful 1st touch pass	0.128	2.708	0.324	0.198	0.514	0.859	0.284	0.072	0.310	1.224	0.401	0.100
Successful tackle	0.023 *	6.962	0.671	0.388	0.071	2.164	0.667	0.164	0.313	1.219	0.400	0.100
Technical action·min^−1^	0.169	2.164	0.270	0.164	0.672	0.637	0.215	0.055	0.867	0.371	0.138	0.033
Unsuccessful tackle	0.652	0.214	0.071	0.019	0.323	1.196	0.392	0.098	0.780	0.493	0.172	0.043
Lost possession (miscontrol)	0.112	2.983	0.351	0.231	0.227	1.434	0.467	0.115	0.462	1.000	0.329	0.083
Total possession per bout	0.982	0.001	0.050	0.000	0.146	1.719	0.551	0.135	0.197	1.526	0.495	0.122

* Significant (*p* < 0.05) main effect of condition.
